# Prevalence, demographic, socio-economic, and lifestyle factors associated with cigarette, e-cigarette, and dual use: evidence from the 2017–2021 Scottish Health Survey

**DOI:** 10.1007/s11739-024-03716-2

**Published:** 2024-07-18

**Authors:** Yusuff Adebayo Adebisi, Duaa Abdullah Bafail, Oluwatobi Ebenezer Oni

**Affiliations:** 1 College of Social Sciences, University of Glasgow, Glasgow, UK; 2 Department of Clinical Pharmacology, Faculty of Medicine, King Abdulaziz University, Jeddah, Saudi Arabia; 3 School of Health and Well-Being, University of Glasgow, Glasgow, UK

**Keywords:** Smoking, Demographic, Socio-economic, Lifestyle factors, Electronic cigarette, Scotland

## Abstract

Understanding the correlation between demographic, socio-economic, and lifestyle factors with e-cigarette use, cigarette smoking, and dual use is essential for targeted public health interventions. This study examines the prevalence of these behaviors in Scotland and identifies the associated factors. We conducted a repeated cross-sectional analysis of the Scottish Health Survey data from 2017 to 2021, leveraging data from 12,644 participants aged 16 and older: 2271 cigarette smokers, 687 e-cigarette users, 428 dual users, and 9258 never users. Weighted prevalences were calculated by age group, sex, and survey year, followed by weighted multinomial logistic regression to explore associated factors. The overall prevalences were 72.0% (95% CI 70.9–73.1) for never users, 18.9% (95% CI 17.9–19.9) for cigarette smokers, 5.5% (95% CI 5.0–6.1) for e-cigarette users, and 3.6% (95% CI 3.2–4.0) for dual users. From 2017 to 2021, cigarette smoking declined from 21.7% (95% CI 19.6–23.9) to 13.1% (95% CI 11.5–15.0), e-cigarette use from 6.5% (95% CI 5.4–7.8) to 4.8% (95% CI 3.6–6.4), and dual use from 3.7% (95% CI 2.9–4.6) to 2.7% (95% CI 1.9–3.7). Age was a critical factor, with the 25–34 age group more likely to use e-cigarettes (*p = *0.007) and the 35–44 age group more likely to engage in dual use (*p = *0.006) compared to the 16–24 age group. Males had higher odds of e-cigarette use than females (*p = *0.031). White individuals had higher odds of using e-cigarettes (*p = *0.023) and being dual users (*p = *0.017) compared to non-whites. Previously married individuals had higher odds of dual use than singles (*p = *0.031). Larger household sizes were linked to reduced odds of all three behaviors (*p = *0.001). Rural residents were less likely to use e-cigarettes compared to urban residents (*p = *0.025). Higher education correlated with lower odds of all three behaviors (*p = *0.001). Manual occupation increased the likelihood of dual use (*p = *0.042). Lower income and higher deprivation significantly increased the odds of all three behaviors (*p* < 0.001). Excessive alcohol consumption was associated with increased odds of the three behaviors (*p* < 0.001). Poor sleep quality correlated with increased odds of dual use (*p = *0.002) and cigarette smoking (*p* < 0.001). Adherence to physical activity guidelines was associated with reduced odds of all three behaviors (cigarette smoking p < 0.001, e-cigarette use *p = *0.031, dual use *p = *0.016). In conclusion, this study showed a decline in the prevalence of cigarette smoking, e-cigarette use, and dual usage from 2017 to 2021 in Scotland. Significant associations with demographic, socio-economic, and lifestyle factors highlight the need for targeted public health interventions.

## Introduction

Recent years have witnessed a significant transformation in the global tobacco landscape, notably marked by the advent and escalating popularity of e-cigarettes. An established consumer product, e-cigarettes impact behaviors and are now being used by over 80 million individuals worldwide as of 2021 [1]. This development has ignited debate within public health communities, particularly regarding their role in smoking cessation as well as long-term safety [2]. In the United Kingdom and across Scotland in particular, e-cigarettes are reshaping traditional tobacco consumption trends [3, 4] and have even been recommended by the government as a safer option to tobacco smoking [5, 6]. Traditional cigarette smoking, responsible for over 8 million global deaths annually [7], now coexists with e-cigarette usage, introducing complex dynamics in smoking behaviors. According to the National Records of Scotland, there were an estimated 685,256 adult smokers in Scotland in 2022, with the Office for National Statistics reporting a smoking prevalence of 13.9%, down from 15.4% in 2019 [3]. Approximately, 10–13% of the adult population in Scotland use e-cigarettes, though usage by never-smokers remains rare at around 2.5%, according to the recent data from Smoking Toolkit Study [4].

E-cigarettes, initially received with scepticism, are now critically evaluated for their potential public health impacts [8]. For example, a recent Cochrane review provides high-certainty evidence that e-cigarettes with nicotine are more effective in quitting smoking than nicotine replacement therapies and e-cigarettes without nicotine [9]. This evidentiary base sets the stage for an examination of smoking habits, particularly in a Scottish context where specific trends and public health policies may uniquely influence these behaviors. Notably, a significant policy development is the proposed ban on the sale and supply of single-use e-cigarettes, set to take effect on 1 April 2025 [10]. Such policies underline the dynamic nature of public health interventions in Scotland, poised to distinctly impact smoking and vaping trends.

The phenomenon of "dual use" involving both traditional cigarettes and e-cigarettes, presents new challenges and opportunities for public health strategies. It necessitates the need for clear understanding of the evolving tobacco use patterns, influenced by individual lifestyle choices, socio-economic conditions, and demographic contexts. Scholarly opinions diverge on the phenomenon of dual use; some researchers view it as a transitional stage where smokers gradually switch to exclusive e-cigarette use, thereby refuting the gateway theory (which posits that the initial use of a less harmful drug can lead to later use of more harmful substances) [11–13]. Meanwhile, others interpret this co-use as indicative of the gateway effect of e-cigarettes, potentially leading to sustained nicotine addiction [14–17]. Public health efforts must navigate the challenge of minimizing e-cigarette risks, particularly among youth and non-smokers, while considering their benefits for smoking cessation. In Scotland, understanding the prevalence and factors related to dual use of e-cigarettes is important for informed policymaking, given the country's distinct health landscape and smoking patterns.

Limited research, in peer-reviewed journals, has specifically focused on the Scottish population using pooled data to understand the prevalence, demographic, socio-economic, and lifestyle factors associated with cigarette, e-cigarette, and dual usage. Most existing evidence comes from government reports, charities, and other stakeholders [3, 4]. Additionally, there is a scarcity of studies leveraging pooled data to understand the dual use of cigarettes and e-cigarettes. This study seeks to fill this research void by leveraging data from the Scottish Health Survey (SHeS) spanning 2017–2021 to explore the prevalence and determinants of cigarette and e-cigarette use in Scotland. By doing so, it aims to provide evidence-based insights that can inform public health strategies and policymaking. Our investigation is particularly timely, given the evolving regulatory landscape and its potential impacts on public health outcomes in Scotland.

## Method

### Data source, study design and participants

This study employed a secondary cross-sectional analysis of data from the SHeS, specifically from the years 2017, 2018, 2019, and 2021. A total of 25,128 participants were present in the combined dataset, with annual contributions of 5300 in 2017, 6790 in 2018, 6881 in 2019, and 6157 in 2021. The 2020 SHeS data, affected by the pandemic and categorized as experimental statistics, was excluded due to its lack of comparability with other years. The SHeS, an important tool for public health assessment in Scotland since its annual inception in 2008 (and previously in 1995, 1998, and 2003), aims to provide a representative sample of the general population living in private households in Scotland [18]. It focuses on Scottish adults aged ≥ 16 years and children aged 0–15 years, offering valuable insights into health outcomes, risks, inequalities, and their changes over time [19].

Participants for the surveys are selected through the postcode address file, encompassing most residential addresses in Scotland [20]. Data collection is primarily carried out through computer-assisted personal interviewing in the homes of respondents, with sensitive questions handled via self-completion booklets [20]. Notably, in 2021, due to COVID-19 restrictions, interviews were conducted by telephone, and as a result, no physical measurements were taken [21]. Participants were also requested to permit the linking of their survey responses with their NHS health records [21]. The dataset used in this study is a combined individual-level file, which includes data from all participants across the 2017, 2018, 2019, and 2021 surveys. The adult response rate (≥ 16 years) for the survey in 2017 was 50.0%, 2018 was 50.0%, and 2019 was 49.0%. The adult response rate for the survey held steady at 50.0% in 2017 and 2018, before dipping slightly to 49.0% in 2019. However, the adoption of a new approach in 2021 due to COVID-19 pandemic led to a remarkable increase in engagement, with the survey garnering 4557 responses from adults—2984 via the opt-in sample and 1573 through the knock-to-nudge approach. The overall adult response rate for 2021 was approximately 85.3%, with response rates of 87.0% for the opt-in sample and 82.2% for the knock-to-nudge sample. This marked a significant improvement over the response rates of the preceding years and comparability with previous years [21]. Given the repeated cross-sectional design of the study, different households and individuals were interviewed each year [21]. This dataset amalgamated information from household questionnaires, main individual schedules, and self-completions. The data collection method, sampling, and other methodical approaches (including weighting to enhance comparability across years and representativeness) have been detailed elsewhere [22].

### Study variables

The study's dataset, comprising data from the 2017, 2018, 2019, and 2021 surveys, originally included 25,128 participants. However, our study focused on adults aged 16 and older i.e. individuals under 16 years (*n* = 7161) were excluded. The age threshold of 16 years was chosen for this study to capture early patterns of tobacco and e-cigarette use, as this age represents the onset of adulthood in Scotland, despite the legal age for tobacco and e-cigarette use being 18. This allows for the examination of early adult behaviors and their potential impact on later habits and health outcomes. To assess e-cigarette usage, a derived variable, 'Ecigtot16', was utilized based on responses to the question "Do you use an e-cigarette or vaping device at all nowadays?" (options: 1 Yes, 2 No). Responses categorized as 'schedule not obtained' (*n* = 25) and 'not applicable' (*n* = 86) were excluded from this analysis. Similarly, cigarette smoking was evaluated using another derived variable, 'cigst3', based on the question "Do you smoke cigarettes nowadays?" (options: 1 Yes, 2 No). Exclusions were made for responses marked as 'refused' (*n* = 32), 'don’t know' (*n* = 5), 'schedule not obtained' (*n* = 25), and 'not applicable' (*n* = 52).

After these exclusions, the dataset was refined to include complete case responses regarding both cigarette and e-cigarette usage, past or present, or never used. This refinement led to a total of 17,742 participants. Subsequently, a new variable was generated to categorize participants into four groups: exclusive current cigarette smokers, exclusive current e-cigarette users, dual users (those using both cigarettes and e-cigarettes), and never users (those who have never used either product). It is important to note that ex-smokers and former e-cigarette users (5098) were excluded, focusing the analysis solely on current users and never users. The exclusion of ex-smokers and former e-cigarette users from the analysis was a strategic decision aimed at concentrating the study's focus on current public health concerns, specifically the active patterns and impacts of smoking and vaping, which are more directly relevant to ongoing public health interventions and policymaking. The final categorization resulted in 2271 exclusive cigarette smokers, 687 exclusive e-cigarette users, 428 dual users, and 9,258 never users, with total participants of 12,644 [See Table 1 for concise summary of participants exclusions to derive analysis population]. Exclusive current cigarette smokers are those who currently smoke cigarettes but do not use e-cigarettes. Exclusive current e-cigarette users are those who currently use e-cigarettes but do not smoke cigarettes. Dual users are those who currently use both cigarettes and e-cigarettes. Never users are those who have never used either product.

**Table 1:** Participants exclusions to derive analysis population

Step	Description	Number excluded	Remaining participants
Step 1	Total participants from 2017, 2018, 2019, and 2021 SHeS datasets	–	25,128
Step 2	Exclude participants under age 16	7161	17,967
Step 3	Exclude participants with incomplete responses for e-cigarette usage	111	17,856
Step 4	Exclude participants with incomplete responses for cigarette smoking	114	17,742
Step 5	Exclude former smokers and former e-cigarette users and missing values	5098	12,644 (final study population)
Step 6	Generate new variable and categorize participants	–	- Current cigarette smokers (*n* = 2271) - Current e-cigarette users (*n* = 687) - Dual users (*n* = 428) - Never users (*n* = 9258)

A comprehensive approach was adopted to identify key demographic, socio-economic, and lifestyle factors, guided by theoretical understanding and the availability of relevant data. Demographic variables included age, which was segmented into 10-year bands (16–24, 25–34, 35–44, 45–54, 55–64, 65–74, 75 +); sex, categorized as male or female; and ethnicity, initially detailed as white-Scottish, white-British, white-other, and then regrouped into White, and Asian and other minority ethnic groups regrouped as Non-White. The simplification of ethnic categories into 'White' and 'Non-White' was undertaken to facilitate a more streamlined analysis. Additionally, residence was classified as Rural or Urban, marital status was divided into single, married/in a relationship and previously married (including separated, divorced, and widowed), and household size was categorized, with 1–3 members considered a small household and 4 or more as a large household, reflecting the average nuclear household in the UK.

For socio-economic status, several variables were considered. Educational level was categorized into post-secondary education (including PhD, Masters, Bachelors, Higher National Certificate/Diploma), secondary education (Scottish standard grade), and a third category for no qualifications or others. Social class was divided into manual (encompassing unskilled, semi-skilled, and skilled manual work) and non-manual (including professional, managerial, technical, and skilled non-manual roles). The Scottish index of multiple deprivation was categorized into quintiles: most deprived, 2, 3, 4, and least deprived. Income level was estimated using the Organisation for Economic Co-operation and Development method, which adjusts household income to account for size and composition, [23] allowing a fairer comparison of living standards. This income data was divided into quintiles (top, 2nd, 3rd, 4th, bottom).

Lastly, lifestyle factors were assessed. Alcohol consumption was classified (based on the UK Chief Medical Officer (CMO) 2016 low-risk guidelines) into categories: never-drinker (individuals who have never consumed alcohol), ex-drinker (individuals who no longer consume alcohol), drink above recommendation (individuals who consume > 14 units of alcohol per week, exceeding low-risk guidelines), and drink within recommendation (individuals who consume alcohol but stay within the recommended limit of up to 14 units per week) [24]. Sleep quality was evaluated with a question about recent sleep loss, categorizing responses as good, moderate, or poor sleep quality. Physical activity was assessed (based on the 2011 CMO time recommendations) categorizing individuals into low activity (engaging in < 150 min of moderate-intensity or < 75 min of vigorous-intensity activity per week), moderate activity (engaging in some activity but not meeting the full recommended levels), or meeting recommended activity level (engaging in at least 150 min of moderate-intensity or 75 min of vigorous-intensity activity per week, or an equivalent combination) [25].

A separate category was established for non-responses, including “not applicable,” “missing value,” “don't know,” “refused,” and “schedule not obtainable.” This category was applied to variables such as income level, alcohol consumption, sleep quality, and ethnicity, as well as cases where data on rural/urban residence were not available.

### Statistical analyses

We presented unweighted descriptive statistics for categorical variables as percentages to show the distribution of the independent variables (comprising key demographic, socio-economic, and lifestyle factors) across the dependent variables (exclusive cigarette use, exclusive e-cigarette use, dual use, and never user). The relationship between the independent variables and the dependent variables was initially explored using the chi-square test of independence for categorical variables. We then determined the weighted prevalence of exclusive cigarette use, exclusive e-cigarette use, dual use, and never user across various age groups, sexes, and year of the survey. This approach provided insights into specific prevalence trends within the combined dataset spanning 2017, 2018, 2019, and 2021.

To optimize variable selection for our regression model, we employed the Least Absolute Shrinkage and Selection Operator (LASSO) technique. LASSO is particularly effective in epidemiological contexts for reducing model complexity and mitigating overfitting by penalizing the absolute size of the regression coefficients. Variables with a non-zero coefficient (threshold set at 0.0004475) in the LASSO model were considered significant and included in subsequent analyses. We then employed multinomial logistic regression to explore how various demographic, socio-economic, and lifestyle factors—including age group, sex, ethnicity, residence, marital status, household size, education level, social class, income level, the Scottish index of multiple deprivation, alcohol use, sleep quality, and physical activity—were associated with smoking behaviors, categorized into exclusive cigarette use, exclusive e-cigarette use, dual use, and non-use. Our model comprehensively incorporated these variables to assess their independent contributions to smoking behavior, controlling for the potential influence of each on the others. This approach allowed us to delineate the specific impact of each factor, ensuring our findings reflect their true association with smoking behaviors without conflating their effects. The results from the multinomial logistic regression were reported as adjusted odds ratios, confidence intervals, and *p* values. This regression model was weighted to adjust for non-response and sampling unit bias *[svyset [pweight* = *int17181921wt], psu(psu) strata (Strata)]*, enhancing comparability with previous years as well as representativeness. This was essential to maintain the integrity and clarity of the data analysis, ensuring that these distinct types of non-responses did not skew the results or interpretations of the key demographic, socio-economic, and lifestyle variables being studied. Information on the weighting approach for the combined adult survey across 2017, 2018, 2019, and 2021 have been detailed elsewhere [21]. The year of survey data collection was also included as a covariate to account for annual variations in the data. We conducted a multicollinearity assessment using the variance inflation factor (VIF), ensuring the stability and reliability of our regression model (minimum VIF = 1.04, maximum VIF = 1.89, mean VIF = 1.53). A *p* value threshold of < 0.05 was employed to determine statistical significance. All statistical analyses were performed using STATA version 18.

## Results

This study examines the characteristics of 12,644 participants, representing approximately 70.3% of the eligible participants aged 16 and older. These participants are categorized into four groups: never users (*n* = 9258), cigarette smokers (*n* = 2271), e-cigarette users (*n* = 687), and dual users (*n* = 428). Significant demographic, socio-economic, and lifestyle differences were observed across groups, as indicated by chi-square tests (See Table 2).

**Table 2:** Relevant characteristics by never users, cigarette smokers, e-cigarette users, and dual users status

Characteristics	Never user (*n* = 9258)	Cigarette smoker (*n* = 2271)	E-cigarette user (*n* = 687)	Dual user (*n* = 428)	All (*N* = 12,644)	χ2, *p* value
Demographic factors						
Age group, years, *n* (%)						
16–24	767 (8.3)	167 (7.4)	40 (5.8)	31 (7.2)	1005 (8.0)	χ2 = 292.5, *p* < 0.001
25–34	1152 (12.4)	377 (16.6)	113 (16.5)	67 (15.7)	1709 (13.5)	
35–44	1290 (13.9)	381 (16.8)	119 (17.3)	85 (20.0)	1875 (14.8)	
45–54	1513 (16.3)	462 (20.3)	164 (23.9)	109 (25.5)	2248 (17.8)	
55–64	1755 (19.0)	444 (19.6)	146 (21.3)	84 (19.6)	2429 (19.2)	
65–74	1662 (18.0)	306 (13.5)	91 (13.3)	42 (9.8)	2101 (16.6)	
75 +	1119 (12.1)	134 (5.9)	14 (2.0)	10 (2.3)	1277 (10.1)	
Sex, *n* (%)						
Male	3798 (41.0)	1055 (46.5)	308 (44.8)	187 (43.7)	5348 (42.3)	χ2 = 24.4, *p* < 0.001
Female	5460 (59.0)	1216 (53.5)	379 (55.2)	241 (56.3)	7296 (57.7)	
Ethnicity, *n* (%)						
White	8,793 (94.9)	2,216 (97.6)	677 (98.5)	423 (98.8)	12,109 (95.7)	χ2 = 61.1, *p* < 0.001
Non-white	461 (4.9)	53 (2.3)	8 (1.2)	4 (0.9)	526 (4.2)	
Missing	4 (0.1)	2 (0.1)	2 (0.3)	1 (0.2)	9 (0.1)	
Residence, *n* (%)						
Rural	572 (6.2)	52 (2.3)	17 (2.5)	12 (2.8)	653 (5.1)	χ2 = 200.2, *p* < 0.001
Urban	2002 (21.6)	296 (13.0)	106 (15.4)	57 (13.3)	2461 (19.5)	
Not available	6684 (72.2)	1923 (84.7)	564 (82.1)	359 (83.9)	9530 (75.4)	
Marital status, *n* (%)						
Married/in a relationship	6092 (65.8)	1067 (47.0)	405 (59.0)	212 (49.5)	7776 (61.5)	χ2 = 309.8, *p* < 0.001
Single	1699 (18.4)	684 (30.1)	154 (22.4)	129 (30.1)	2666 (21.1)	
Previously married	1466 (15.8)	520 (22.9)	128 (18.6)	87 (20.3)	2201 (17.4)	
Household size, *n* (%)						
Small (1–3)	7418 (80.1)	1899 (83.6)	569 (82.8)	369 (86.2)	10,255 (81.1)	χ2 = 23.8, *p* < 0.001
Large (4 +)	1840 (19.9)	372 (16.4)	118 (17.2)	59 (13.8)	2389 (18.9)	
Socio-economic factors						
Education level, *n* (%)						
Post-secondary	5188 (56.0)	656 (28.9)	293 (42.7)	141 (32.9)	6278 (49.7)	χ2 = 691.2, *p* < 0.001
Secondary education	3020 (32.6)	1023 (45.1)	286 (41.6)	197 (46.0)	4526 (35.8)	
No formal qualification	1037 (11.2)	585 (25.8)	105 (15.3)	89 (20.8)	1816 (14.4)	
Missing	13 (0.2)	7 (0.3)	3 (0.4)	1 (0.2)	24 (0.2)	
Social class, *n* (%)						
Manual occupation	2556 (27.6)	1286 (56.6)	311 (45.3)	215 (50.2)	4368 (34.6)	χ2 = 789.2, *p* < 0.001
Non-manual occupation	6133 (66.3)	857 (37.7)	354 (51.5)	187 (43.7)	7531 (59.6)	
Missing	569 (6.2)	128 (5.6)	22 (3.2)	26 (6.1)	745 (5.8)	
Income level, *n* (%)						
Top quintile	1964 (21.2)	213 (9.4)	108 (15.7)	45 (10.5)	2330 (18.4)	χ2 = 503.6, *p* < 0.001
2nd quintile	1763 (19.0)	301 (13.3)	124 (18.1)	55 (12.9)	2243 (17.7)	
3rd quintile	1510 (16.3)	328 (14.4)	117 (17.0)	69 (16.1)	2024 (16.0)	
4th quintile	1410 (15.2)	423 (18.6)	121 (17.6)	74 (17.3)	2028 (16.0)	
Bottom quintile	1111 (12.0)	588 (25.9)	134 (19.5)	113 (26.4)	1946 (15.4)	
Missing	1500 (16.2)	418 (18.4)	83 (12.1)	72 (16.8)	2073 (16.4)	
Scottish index of multiple deprivation, *n* (%)						
Most deprived	1098 (11.9)	701 (30.9)	164 (23.9)	144 (33.6)	2107 (16.7)	χ2 = 972.5, *p* < 0.001
2	1596 (17.2)	566 (24.9)	193 (28.1)	111 (25.9)	2466 (19.5)	
3	1951 (21.1)	448 (19.7)	150 (21.8)	69 (16.1)	2618 (20.7)	
4	2313 (25.0)	331 (14.6)	93 (13.5)	63 (14.7)	2800 (22.1)	
Least deprived	2300 (24.8)	225 (9.9)	87 (12.7)	41 (9.6)	2653 (21.0)	
Lifestyle factors						
Alcohol use, *n* (%)						
Never-drinker	886 (9.6)	121 (5.3)	20 (2.9)	22 (5.1)	1049 (8.3)	χ2 = 317.4, *p* < 0.001
Ex-drinker	743 (8.0)	327 (14.4)	82 (11.9)	58 (13.6)	1210 (9.6)	
Drink above recommendation	2971 (32.1)	962 (42.4)	291 (42.4)	179 (41.8)	4403 (34.8)	
Drink below recommendation	4534 (49.0)	818 (36.0)	280 (40.8)	164 (38.3)	5796 (45.8)	
Missing	124 (1.3)	43 (1.9)	14 (2.0)	5 (1.2)	186 (1.5)	
Sleep quality, *n* (%)						
Poor	222 (2.4)	150 (6.6)	34 (5.0)	37 (8.6)	443 (3.5)	χ2 = 207.9, *p* < 0.001
Moderate	1040 (11.2)	334 (14.7)	77 (11.2)	73 (17.1)	1524 (12.1)	
Good	6800 (73.5)	1430 (63.0)	484 (70.5)	252 (58.9)	8,966 (70.9)	
Missing	1196 (12.9)	357 (15.7)	92 (13.4)	66 (15.4)	1711 (13.5)	
Physical activity, *n* (%)						
Low	2568 (27.7)	835 (36.8)	200 (29.1)	150 (35.1)	3,753 (29.7)	χ2 = 84.9, *p* < 0.001
Moderate	1084 (11.7)	231 (10.2)	86 (12.5)	53 (12.4)	1454 (11.5)	
Meets recommendation	5595 (60.4)	1198 (52.8)	400 (58.2)	224 (52.3)	7417 (58.7)	
Missing	11 (0.12)	7 (0.3)	1 (0.2)	1 (0.2)	20 (0.2)	

Statistically significant *p* value < 0.05

Age distribution varied significantly among the groups (*p* < 0.001), with a lower percentage of younger participants (16–24 years) among e-cigarette users (5.8%) compared to other groups. Notably, the proportion of participants aged 45–54 was highest among dual users (25.5%). Gender differences were also significant (*p* < 0.001), with a lower percentage of males in the three categories and a higher female population in the study. Ethnicity showed notable disparities (*p* < 0.001), with white participants dominating all categories, particularly among dual users (98.8%). Education level varied greatly (*p* < 0.001), with post-secondary education being less common among cigarette smokers (28.9%) compared to never users (56.0%). Social class and income level differences were pronounced (*p* < 0.001). The Scottish Index of Multiple Deprivation revealed a significant trend (*p* < 0.001), with the most deprived category being overrepresented among cigarette smokers (30.9%) and dual users (33.6%). Alcohol consumption patterns differed significantly across groups (*p* < 0.001), with cigarette smokers and dual users more likely to drink above recommended levels. Sleep quality was poorest among dual users (8.6% reporting poor sleep, *p* < 0.001). Physical activity levels showed that a larger proportion of never users met the recommended levels (60.4%), compared to cigarette smokers (52.8%) and dual users (52.3%), with a significant difference across groups (*p* < 0.001).

**Fig. 1:**
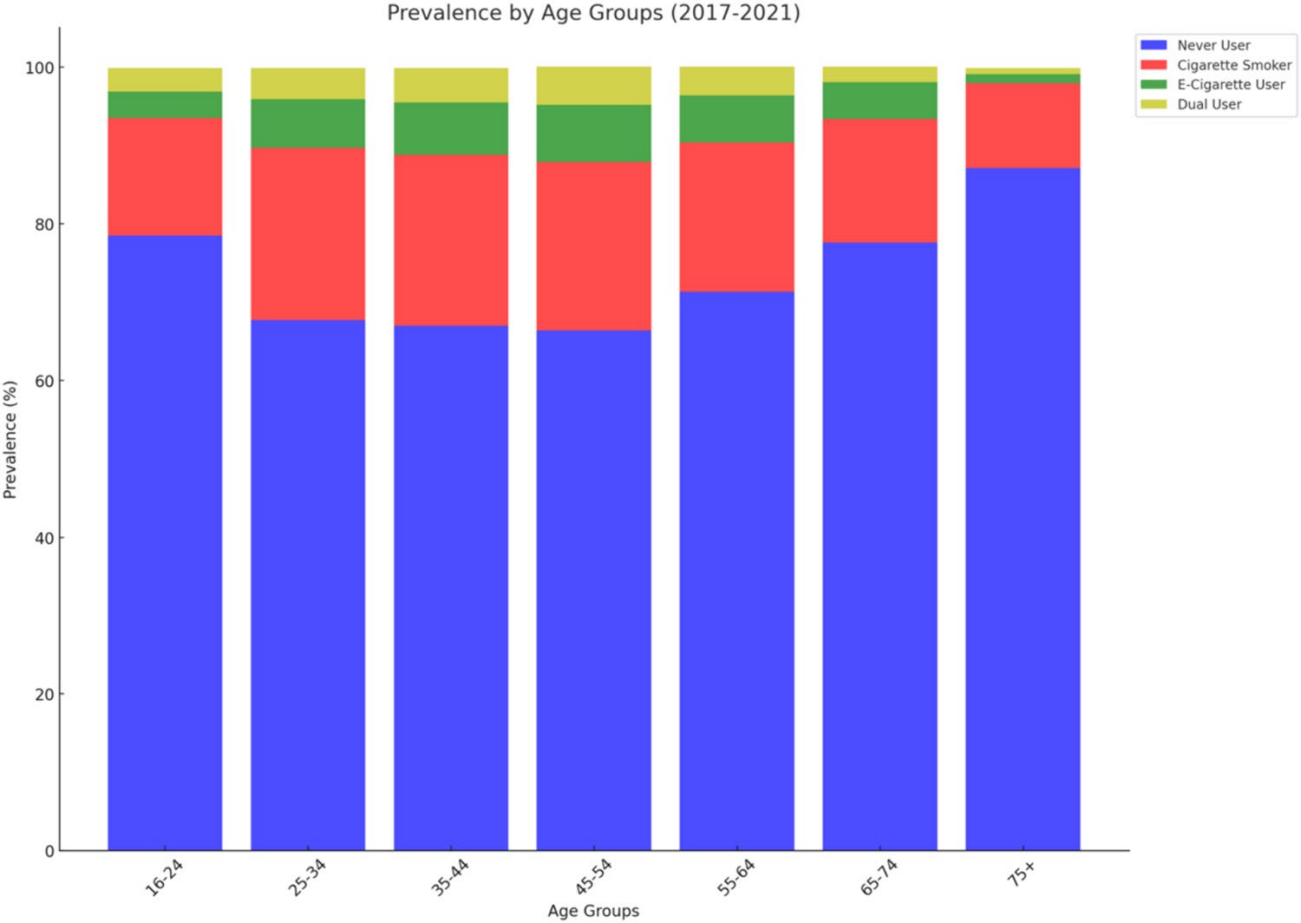
Prevalence of never users, cigarette smokers, e-cigarette users, and dual users’ status by age groups, 2017–2021

In Table 3, this analysis presented the pooled weighted prevalence of never users, cigarette smokers, e-cigarette users, and dual users across various age groups, by sex and survey year, covering the period from 2017 to 2021. The overall prevalence among the study population were 72.0% (95% CI 70.9–73.1) for never users, 18.9% (95% CI 17.9–19.9) for cigarette smokers, 5.5% (95% CI 5.0–6.1) for e-cigarette users, and 3.6% (95% CI 3.2–4.0) for dual users. The prevalence of never users was highest among the 75 + age group at 87.1% (95% CI 84.9–89.1) and lowest among the 45–54 age group at 66.4% (95% CI 63.8–68.9). Cigarette smoking prevalence peaked in the 25–34 age group at 22.0% (95% CI 19.6–24.6) and was lowest in the 75 + age group at 10.9% (95% CI 9.1–13.0). E-cigarette use showed an increasing trend with age up to 45–54 years, where it reached its peak prevalence of 7.3% (95% CI 6.1–8.7), then declined. The prevalence of dual users was highest in the 45–54 age group at 4.9% (95% CI 3.9–6.1) and notably decreased with age, showing the lowest prevalence in the 75 + age group at 0.8% (95% CI 0.4–1.6). See Fig. 1 for visualized plot for prevalence by age groups.

**Table 3:** Pooled weighted prevalence of never users, cigarette smokers, e-cigarette users, and dual users status across age group, sex and survey year, 2017–2021

Characteristics	Never user	Cigarette smoker	E-cigarette user	Dual user
Prevalence, % (95% CI)	72.0 (70.9–73.1)	18.9 (17.9–19.9)	5.5 (5.0–6.1)	3.6 (3.2–4.0)
Age group, % (95% CI)				
16–24	78.5 (75.0–81.8)	15.0 (12.2–18.2)	3.4 (2.2–5.4)	3.0 (2.0–4.6)
25–34	67.7 (64.9–70.4)	22.0 (19.6–24.6)	6.2 (4.9–7.8)	4.0 (3.0–5.4)
35–44	67.0 (64.1–69.8)	21.8 (19.3–24.6)	6.7 (5.4–8.4)	4.4 (3.5–5.5)
45–54	66.4 (63.8–68.9)	21.5 (19.3–23.8)	7.3 (6.1–8.7)	4.9 (3.9–6.1)
55–64	71.3 (69.1–73.3)	19.1 (17.2–21.1)	6.0 (5.0–7.1)	3.7 (2.9–4.7)
65–74	77.6 (75.3–79.6)	15.8 (14.0–17.8)	4.7 (3.7–6.0)	2.0 (1.4–2.7)
75 +	87.1 (84.9–89.1)	10.9 (9.1–13.0)	1.1 (0.6–2.0)	0.8 (0.4–1.6)
Sex, % (95% CI)				
Male	69.3 (67.7–70.9)	20.8 (19.4–22.3)	6.1 (5.3–7.0)	3.8 (3.2–4.5)
Female	74.5 (73.2–75.8)	17.1 (16.1–18.3)	5.0 (4.5–5.6)	3.3 (2.9–3.8)
Survey year, % (95% CI)				
2017	68.2 (65.6–70.6)	21.7 (19.6–23.9)	6.5 (5.4–7.8)	3.7 (2.9–4.6)
2018	69.9 (67.5–72.1)	20.8 (18.7–23.0)	4.9 (4.1–5.9)	4.4 (3.6–5.3)
2019	70.4 (68.3–72.4)	20.2 (18.3–22.2)	6.0 (5.1–7.0)	3.4 (2.8–4.3)
2021	79.4 (76.9–81.7)	13.1 (11.5–15.0)	4.8 (3.6–6.4)	2.7 (1.9–3.7)

**Fig. 2:**
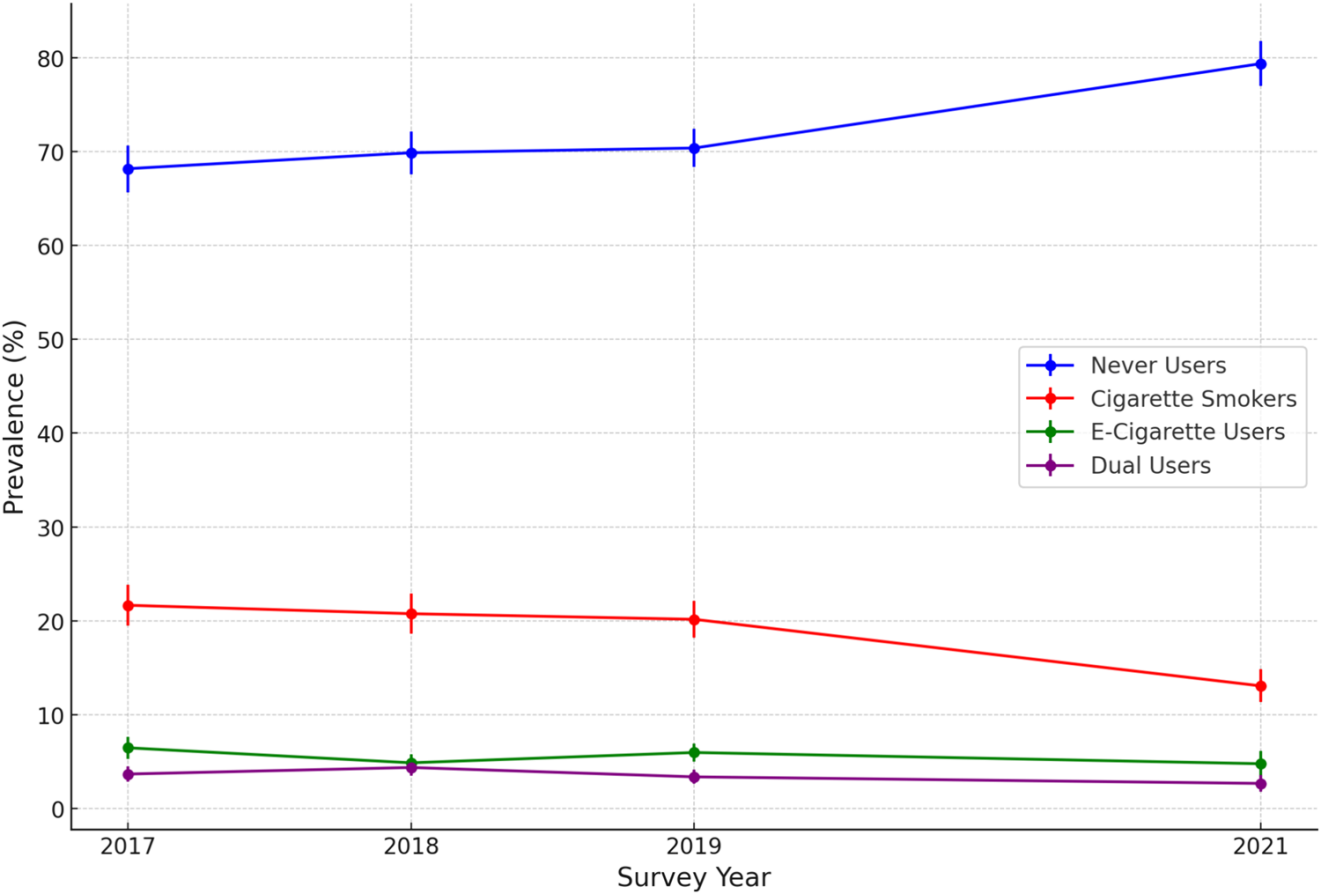
Prevalence of never users, cigarette smokers, e-cigarette users, and dual users’ status by year, 2017–2021

Males exhibited higher prevalence of cigarette smoking (20.8%, 95% CI 19.4–22.3), e-cigarette use (6.1%, 95% CI 5.3–7.0), and dual use (3.8%, 95% CI 3.2–4.5) compared to females. Between 2017 and 2021, the prevalence of never users significantly increased from 68.2% (95% CI 65.6–70.6) to 79.4% (95% CI 76.9–81.7), while cigarette smoking declined from 21.7% (95% CI 19.6–23.9) to 13.1% (95% CI 11.5–15.0), and dual use decreased from 3.7% (95% CI 2.9–4.6) to 2.7% (95% CI 1.9–3.7), all indicating significant shifts in smoking behaviors (See Fig. 2). E-cigarette use slightly decreased from its peak of 6.5% (95% CI 5.4–7.8) in 2017 to 4.8% (95% CI 3.6–6.4) in 2021.

Our multivariable analysis in Table 4 revealed significant associations across demographic, socio-economic, and lifestyle factors with e-cigarette use, dual use, and cigarette smoking.

**Table 4:** Demographic, socio-economic and lifestyle factors associated with cigarette smokers, e-cigarette users, and dual users status using weighted multinomial logistic regression

Variables	Never smoker (base outcome)		
	Cigarette smoker AOR (95% CI), *p* value	E-cigarette user AOR (95% CI), *p* value	Dual user AOR (95% CI), *p* value
Demographic factors			
Age group, years			
16–24	Reference	Reference	Reference
25–34	2.49 (1.80–3.44), *p* < 0.001	2.21 (1.24–3.94), *p* = 0.007	1.82 (0.99–3.32), *p* = 0.050
35–44	2.59 (1.91–3.52), *p* < 0.001	2.59 (1.46–4.59), *p* = 0.001	2.20 (1.25–3.86), *p* = 0.006
45–54	2.12 (1.55–2.90), *p* < 0.001	2.47 (1.37–4.45), *p* = 0.003	1.91 (1.09–3.34), *p* = 0.023
55–64	1.27 (0.90–1.78), *p* = 0.170	1.44 (0.77–2.68), *p* = 0.255	0.93 (0.52–1.65), *p* = 0.795
65–74	0.71 (0.50–1.02), *p* = 0.062	0.86 (0.44–1.66), *p* = 0.644	0.33 (0.17–0.65), *p* = 0.001
75 +	0.31 (0.21–0.46), *p* < 0.001	0.16 (0.07–0.38), *p* < 0.001	0.09 (0.04–0.23), *p* < 0.001
Sex			
Male	1.17 (1.02–1.32), *p* = 0.016	1.22 (1.02–1.47), *p* = 0.031	1.20 (0.96–1.51), *p* = 0.112
Female	Reference	Reference	Reference
Ethnicity			
White	1.91 (1.30—2.82), *p* = 0.001	3.07 (1.17—8.05), *p* = 0.023	5.59 (1.35—23.1), *p* = 0.017
Non-white	Reference	Reference	Reference
Residence			
Rural	1.17 (0.77–1.79), *p* = 0.456	0.47 (0.24–0.91), *p* = 0.025	1.04 (0.45–2.40), *p* = 0.921
Urban	Reference	Reference	Reference
Married status			
Married/in a relationship	0.71 (0.60–0.85), *p* < 0.001	0.94 (0.72–1.23), *p* = 0.671	0.87 (0.63–1.20), *p* = 0.396
Single	Reference	Reference	Reference
Previously married	1.30 (1.06–1.59), *p* = 0.012	1.31 (0.96–1.81), *p* = 0.088	1.52 (1.04–2.23), *p* = 0.031
Household size			
Small (1–3)	Reference	Reference	Reference
Large (4 +)	0.72 (0.59–0.88), *p* = 0.001	0.61 (0.45–0.81), *p* = 0.001	0.44 (0.31–0.62), *p* = 0.001
Socio-economic factors			
Education level			
Post-secondary	0.28 (0.23–0.35), *p* < 0.001	0.58 (0.41–0.80), *p* = 0.001	0.39 (0.27–0.58), *p* = 0.001
Secondary education	0.51 (0.43–0.62), *p* < 0.001	0.72 (0.53–0.98), *p* = 0.037	0.64 (0.46–0.89), *p* = 0.010
No formal qualification	Reference	Reference	Reference
Social class			
Manual occupation	1.93 (1.68–2.22), *p* < 0.001	1.36 (1.10–1.69), *p* = 0.005	1.36 (1.01–1.83), *p* = 0.042
Non-manual occupation	Reference	Reference	Reference
Income level			
Top quintile	0.35 (0.27–0.44), *p* < 0.001	0.43 (0.30–0.61), *p* < 0.001	0.31 (0.19–0.50), *p* < 0.001
2nd quintile	0.45 (0.35–0.58), *p* < 0.001	0.57 (0.42–0.79), *p* = 0.001	0.37 (0.24–0.59), *p* < 0.001
3rd quintile	0.55 (0.44–0.70), *p* < 0.001	0.61 (0.44–0.85), *p* = 0.003	0.56 (0.37–0.86), *p* = 0.007
4th quintile	0.61 (0.49–0.76), *p* < 0.001	0.71 (0.54–0.94), *p* = 0.017	0.61 (0.41–0.90), *p* = 0.012
Bottom quintile	Reference	Reference	Reference
Scottish index of multiple deprivation			
Most deprived	2.73 (2.12–3.51), *p* < 0.001	2.28 (1.52–3.41), *p* < 0.001	3.36 (2.12–5.35), *p* < 0.001
2	1.96 (1.56–2.47), *p* < 0.001	2.19 (1.50–3.19), *p* < 0.001	2.50 (1.60–3.93), *p* < 0.001
3	1.65 (1.29–2.13), *p* < 0.001	1.54 (1.06–2.24), *p* = 0.024	1.38 (0.83–2.29), *p* = 0.215
4	1.26 (0.97–1.63), *p* = 0.080	0.95 (0.63–1.44), *p* = 0.821	1.36 (0.82–2.26), *p* = 0.240
Least deprived	Reference	Reference	Reference
Lifestyle factors			
Alcohol use			
Never-drinker	Reference	Reference	Reference
Ex-drinker	3.18 (2.33–4.34), *p* < 0.001	4.14 (2.20–7.80), *p* < 0.001	2.72 (1.39–5.32), *p* = 0.003
Drink above recommendation	4.42 (3.27–5.96), *p* < 0.001	4.88 (2.57–9.26), *p* < 0.001	3.17 (1.76–5.69), *p* < 0.001
Drink within recommendation	1.96 (1.47–2.61), *p* < 0.001	2.69 (1.48–4.89), *p* = 0.001	1.68 (0.95–2.98), *p* = 0.073
Sleep quality			
Poor	1.49 (1.07–2.06), *p* < 0.001	1.29 (0.83–1.99), *p* = 0.261	2.21 (1.35–3.65), *p* = 0.002
Moderate	1.39 (1.15–1.67), *p* = 0.019	0.94 (0.70–1.26), *p* = 0.681	1.48 (1.07–2.04), *p* = 0.019
Good	Reference	Reference	Reference
Physical activity			
Low	Reference	Reference	Reference
Moderate	0.85 (0.68–1.05), *p* = 0.133	1.08 (0.80–1.44), *p* = 0.614	0.97 (0.66–1.41), *p* = 0.855
Meets recommendation	0.70 (0.60–0.81), *p* < 0.001	0.77 (0.60–0.98), *p* = 0.031	0.70 (0.52–0.93), *p* = 0.016

Statistically significant *p* value < 0.05

Variables are mutually adjusted in the multivariable model

Age significantly impacts e-cigarette use. Individuals aged 25–34 have higher odds of e-cigarette use (AOR: 2.21, 95% CI 1.24–3.94, *p* = 0.007) compared to those aged 16–24. Gender analysis highlighted that males are more likely to use e-cigarettes (AOR: 1.22, 95% CI 1.02–1.47, *p* = 0.031) compared to females. Ethnicity plays a substantial role, with White individuals having considerably higher odds of e-cigarette use (AOR: 3.07, 95% CI 1.17–8.05, *p* = 0.023) than non-White individuals. Individuals residing in rural areas were less likely to use e-cigarettes (AOR: 0.47, 95% CI 0.24–0.91, *p* = 0.025) compared to those in urban settings. Socio-economic status, as indicated by educational level, showed that individuals with post-secondary education are less likely to engage in e-cigarette use (AOR: 0.58, 95% CI 0.41–0.80, *p* = 0.001) compared to those with no formal qualification. Compared to individuals in the bottom income quintile, those in the top quintile had lower odds of being e-cigarette users (AOR: 0.43, 95% CI 0.30–0.61, *p* < 0.001). Lifestyle factors, including alcohol consumption, significantly affect e-cigarette use. Ex-drinkers have increased odds of e-cigarette use (AOR: 4.14, 95% CI 2.20–7.80, *p* < 0.001). Meeting physical activity recommendations is associated with lower odds of e-cigarette use (AOR: 0.77, 95% CI 0.60–0.98, *p* = 0.031).

Age also impacts dual use, with individuals aged 35–44 more likely to be dual users (AOR: 2.20, 95% CI 1.25–3.86, *p* = 0.006). White individuals have higher odds of dual use (AOR: 5.59, 95% CI 1.35–23.1, *p* = 0.017) than non-White individuals. Specifically, previously married individuals had higher odds of dual use (AOR: 1.52, 95% CI 1.04–2.23, *p* = 0.031) compared to those who were single. Individuals with post-secondary education are less likely to engage in dual use (AOR: 0.39, 95% CI 0.27–0.58, *p* = 0.001) compared to those with no formal qualification. Manual occupation was associated with increased odds of dual use (AOR: 1.36, 95% CI 1.01–1.83, *p* = 0.042) compared to non-manual occupations. Compared to individuals in the bottom income quintile, those in the top quintile had lower odds of being dual users (AOR: 0.31, 95% CI 0.19–0.50, *p* < 0.001). The most deprived individuals had significantly higher odds of dual use (AOR: 3.36, 95% CI 2.12–5.35, *p* < 0.001) compared to the least deprived. Lifestyle factors, including alcohol consumption, significantly affect dual use. Ex-drinkers have increased odds of dual use (AOR: 2.72, 95% CI 1.39–5.32, *p* = 0.003). Poor sleep quality is correlated with dual use (AOR: 2.21, 95% CI 1.35–3.65, *p* = 0.002). Meeting physical activity recommendations is associated with lower odds of dual use (AOR: 0.70, 95% CI 0.52–0.93, *p* = 0.016).

Compared to individuals in the bottom income quintile, those in the top quintile had lower odds of being cigarette smokers (AOR: 0.35, 95% CI 0.27–0.44, *p* < 0.001). Poor sleep quality is correlated with cigarette smoking (AOR: 1.49, 95% CI 1.07–2.06, *p* < 0.001). Excessive alcohol consumption is associated with increased odds of cigarette smoking (AOR: 4.42, 95% CI 3.27–5.96, *p* < 0.001).

## Discussion

Our research provides a novel examination of the prevalence and factors associated with cigarette smoking, e-cigarette use, and dual usage in Scotland, highlighting distinct patterns of use across different demographic groups using a 4 year cross-sectional surveys. The analysis reveals that a significant portion of the population has never used tobacco products (72.0%), with current cigarette smokers constituting 18.9% of the population. E-cigarette users’ account for 5.5%, while dual users, those who engage in both cigarette smoking and e-cigarette use, make up 3.6%. These figures indicate that within the surveyed population, traditional cigarette smoking remains more prevalent than e-cigarette use. This finding aligns with results from the NHANES database of the US population. In the NHANES survey conducted from 2015 to 2018, out of a total of 266,058 respondents, 9.72% (79,825 respondents) reported using e-cigarettes, and 29.37% reported smoking traditional cigarettes [26]. Similarly, another study using data from the 2015–2018 National Health Interview Surveys (NHIS) of the US population found that among 55,780 adults, 0.4% were current exclusive e-cigarette users (*n* = 185), and 5.8% were current exclusive daily smokers (*n* = 3632) [27].

Our study also revealed variability in tobacco product use across different age groups, suggesting demographic differences in tobacco and e-cigarette consumption patterns [28]. The observed prevalence of dual use, notably higher in the middle-aged group at 4.9% for those aged 45–54, suggests it might not be a long-term behavior but a transitional phase towards potentially using e-cigarettes exclusively, possibly as a method to quit smoking [29]. This idea is further supported by the lower engagement with e-cigarettes among the youngest surveyed group, indicating that e-cigarettes might be more commonly used by existing smokers looking to reduce or quit their habit [11–13, 29]. This perspective aligns with evolving discussions on e-cigarettes as cessation tools [9]. The Smokefree Great Britain analysis conducted by Action on Smoking and Health and YouGov in Spring 2023 found that 9.1% of the British adult population, or 4.7 million people, use e-cigarettes, the highest rate recorded. Of these users, 2.7 million (56%) are ex-smokers, 1.7 million (37%) are current smokers, and 320,000 are never smokers [30]. Studies support this view, showing that e-cigarettes can be an effective tool for smoking cessation among existing smokers. For instance, a recent study found that e-cigarette usage nudged smokers toward quitting, even those who initially had no intention of stopping [31]. In contrast, a systematic review and meta-analysis found a significant uptake of tobacco smoking among non-smokers exposed to e-cigarettes compared with those not exposed [14], highlighting the increasing trend of e-cigarette use among non-smoking youth. Therefore, while e-cigarettes have potential benefits for smoking cessation, their broader impact, particularly on non-smoking youth, requires careful consideration.

While past research has often documented the increase in e-cigarette usage [32], its direct relationship with declining cigarette smoking rates has been less clear. The findings of the current study, which include a breakdown across different age categories and a notable proportion of individuals who have never used tobacco products, provide evidence of lower e-cigarette and dual-use prevalence among both younger and older adults. Our findings are similar to those of the Public Health England report on e-cigarette use, which revealed that regular vaping remains low among young people and has plateaued among adults [33]. This echoes findings from the United States, where it was reported that from 2019 to 2020, the prevalence of overall tobacco product use, combustible tobacco product use, cigarettes, e-cigarettes, and the use of two or more tobacco products decreased [34]. Contrastingly, a global systematic review based on 53 articles and 5 eligibility criteria reported the international pooled prevalence of young people’s current e-cigarette use at 7.7%, and dual use at 4.0% [35]. These figures are higher than those reported in our study, where current e-cigarette use is 3.4% and dual use is 3.0% among those age 16–24. This implies it would be appropriate to emphasize that this prevalence is not yet stable, as the phenomenon is continuously changing.

Additionally, this study also revealed gender differences in tobacco use, with men more likely to smoke cigarettes, use e-cigarettes, and engage in dual use, while women are more often never users. This pattern is consistent with historical trends in tobacco consumption [36]. Our analysis also uncovered a decline in cigarette smoking, e-cigarette use, and dual usage from 2017 to 2021, suggesting the necessity for a thorough temporal investigation to help understand the underlying reasons or factors influencing the observed changes within the Scottish population. Factors such as enhanced public health campaigns, shifts in societal attitudes, and stricter tobacco regulations may have contributed to these trends. While it is clear that the prevalences are declined in our study, the size of the drop between 2019 and 2021 should be treated with caution due to the change in the method of data collection [21]. A deeper understanding of these influences will aid in developing effective strategies to continue reducing tobacco use.

Furthermore, the findings from the weighted multinomial logistic regression analysis also revealed that ethnicity emerged as a significant factor, with individuals from white backgrounds showing higher odds of engaging in cigarette smoking, e-cigarette use, and notably, dual use compared to non-white counterparts, similar to what has been previously reported [37]. This distinction may reflect cultural, socio-economic, or accessibility differences in tobacco products and cessation aids. Urban versus rural residence showed that e-cigarette use was significantly less likely in rural areas, perhaps due to differences in product availability or cultural acceptance. However, this is in contrast with previous studies where no difference was found [38]. Marital status also played a role, with those previously married showing a higher likelihood of dual use, suggesting that life changes or stressors associated with relationship dissolution might influence this behavior [39]. Interestingly, individuals from larger households had lower odds of being cigarette smokers, e-cigarette users, and dual users. This finding could be attributed to the social and familial pressures or responsibilities that discourage tobacco use, or possibly, the financial constraints of supporting a larger household limiting disposable income for tobacco products. Compared with past studies, these findings underline the complexity of smoking behaviors, influenced by a web of socio-demographic factors [40, 41]. Past research often highlighted the impact of socio-economic status and education on smoking prevalence, [42, 43] but the analysis of household size and marital status adds depth to the understanding of how personal circumstances and living arrangements can affect these behaviours.

Furthermore, the regression analysis provides insightful correlations between socio-economic factors—education level, social class, income level, and the Scottish index of multiple deprivation—and cigarette smoking, e-cigarette use, and dual use. Individuals with post-secondary education were significantly less likely to engage in cigarette smoking, e-cigarette use, and dual use compared to those with no formal qualifications, emphasizing the protective effect of higher education, similar to previous findings [44–46]. Similarly, those in manual occupations were more likely to smoke cigarettes or engage in dual use, [47] highlighting occupational influences on tobacco consumption. Income level also showed a clear gradient effect, with individuals in the top income quintile exhibiting the lowest likelihood of cigarette smoking, e-cigarette use, or dual use. This is similar to what has been previously reported [48, 49].

The SIMD findings reveal a clear disparity in tobacco use, with those living in the most deprived areas being significantly more likely to smoke cigarettes, use e-cigarettes, and engage in dual use. This pattern indicates a strong socio-economic gradient, where deprivation amplifies the likelihood of engaging in harmful health behaviors. Compared to previous studies, [48] these findings reaffirm the established link between lower socio-economic status and cigarettes, e-cigarettes, and dual use, but they also offer a more granular understanding of how different socio-economic indicators interact with use patterns. Justifying these associations, the data suggests that socio-economic factors play a key role in shaping individuals' health behaviors [48, 50]. Education, income, and living conditions not only affect individuals' access to resources and health information but is also associated their lifestyle choices. The implications of these findings are significant for public health policy and interventions, advocating for a targeted approach that addresses the underlying socio-economic determinants of health. Interventions aimed at reducing tobacco use must, therefore, not only focus on individual behavior change but also seek to improve the socio-economic conditions that predispose certain populations to higher tobacco use.

The analysis also indicates associations between lifestyle factors—alcohol use, sleep quality, and physical activity—and the use of cigarettes, e-cigarettes, and dual usage. It shows that higher alcohol consumption is linked with an increased likelihood of engaging in cigarette smoking, e-cigarette use, and dual usage. This finding is consistent with existing literature that notes a co-occurrence of alcohol and nicotine use [51]. Poor sleep quality is associated with a greater likelihood of engaging in cigarette smoking and dual usage, similar to what has been previously reported [52]. Conversely, regular physical activity is associated with lower odds of cigarette and e-cigarette use, indicating a possible protective effect of exercise against nicotine use [53]. These findings highlight the relationship of lifestyle choices with nicotine use behaviors, pointing towards the potential benefits of incorporating lifestyle factors into public health strategies aimed at reducing nicotine use. Encouraging physical activity, addressing alcohol consumption, and improving sleep quality could be valuable components of smoking cessation programs [54–57].

The public health implications of our findings reify the need for targeted, evidence-based interventions that address the observed demographic and socio-economic disparities in smoking behaviors. Specifically, the higher prevalence of smoking and vaping among individuals from deprived areas and lower socio-economic backgrounds suggests that interventions should not only focus on behavior change but also tackle the underlying socio-economic determinants of health. This approach could include policies aimed at reducing poverty, improving education, and enhancing access to smoking cessation resources as well as active public health surveillance in these communities. Additionally, considering the significant role of lifestyle factors such as alcohol use and physical activity, comprehensive public health strategies could benefit from incorporating programs that promote healthier lifestyles alongside smoking cessation efforts. In the broader social and policy context, it is essential to align these interventions with Scotland's existing tobacco control policies, ensuring they are responsive to the changing landscape of tobacco and nicotine product use.

This study leverages the extensive data collected by the Scottish Health Survey over several years to conduct an in-depth analysis of smoking behaviors across Scotland. Nonetheless, it is important to acknowledge certain limitations inherent in our approach. Firstly, the cross-sectional design of the study, while offering a broad snapshot of smoking behaviors at specific points in time, inherently limits our ability to infer causality. We suggest that future research adopt a longitudinal design to establish the directional relationships more definitively between variables. Secondly, we acknowledge that the reliance on self-reported smoking data may introduce bias due to social desirability and recall inaccuracies, which could affect the accuracy of the reported smoking behaviors. Efforts to validate self-reported data with objective measures could enhance reliability for future research.

Thirdly, the term "nowadays" used in the survey questions to measure smoking and vaping behaviors can only indicate any current use, which serves as a minimal indicator of current use. This approach does not capture the frequency or intensity of use, nor does it differentiate between daily and occasional use. For example, it does not measure if respondents use cigarettes daily while vaping occasionally, a common pattern of dual use. Future studies should incorporate more detailed questions regarding the frequency and intensity of use to provide a more nuanced understanding of smoking and vaping behaviors. Lastly, the exclusion of the 2020 survey data, a year significantly affected by the pandemic, represents a gap in our analysis, as behavioral changes during this period are not captured. However, evidence regarding whether the pandemic increased smoking behavior is mixed, uncertain and complex [58–60]. Furthermore, using an opt-in method in 2021 led to fewer respondent households from the most deprived areas and fewer respondents from the youngest age group compared to past surveys [21]. However, interviews conducted with the knock-to-nudge sample helped make the overall sample more similar to those of previous years. The application of a weighting strategy aimed to adjust the findings to reflect the entire household population more accurately. Additionally, the inclusion and adjustment for survey year in our regression model aim to mitigate year-to-year variations, albeit not fully compensating for the unique impacts of the pandemic. Future studies should consider the pandemic's potential effects on smoking behaviors to provide a more comprehensive understanding. Additionally, we also acknowledge that our model does not explicitly account for factors such as policy changes, cultural shifts, and regional variations within Scotland, which are important considerations, and we suggest them as areas for future research to provide a more comprehensive understanding of smoking behaviors.

## Conclusion

This study enhances the understanding of the prevalence as well as how demographic, socio-economic, and lifestyle factors intersect with the use of cigarettes, e-cigarettes, and their dual use in Scotland. It reveals that traditional cigarette smoking is more prevalent compared to e-cigarette use and dual usage, with middle-aged adults more frequently engaging in e-cigarette and dual usage compared to the younger adults. The analysis revealed significant demographic and socio-economic disparities that are associated with smoking behaviors, pointing towards the need for tailored public health interventions. Moreover, lifestyle choices, including alcohol consumption, sleep quality, and physical activity, are closely associated with e-cigarette use and dual use, suggesting a complex interplay between various factors.

These findings reify the necessity for comprehensive tobacco control strategies in Scotland that address not just individual behaviors but also broader lifestyle determinants. By highlighting the associations between lifestyle factors and smoking behaviors, this study provides valuable insights for developing risk-appropriate regulations and public health promotion campaigns. These efforts are important to maintaining low prevalence rates of cigarette, e-cigarette, and dual use among younger adults and non-smokers, ultimately contributing to better public health outcomes. Therefore, readers should engage with this study to inform the design and implementation of effective tobacco control and harm reduction measures that consider the complex nature of these behaviors.

## Data Availability

To download the dataset used in the analyses, please visit the https://ukdataservice.ac.uk/find-data/browse/health/.

## Declarations

### Patient and public involvement

Patients and/or the public were not involved in the design, or conduct, or reporting, or dissemination plans of this research.
